# Atypical Sound Perception in ASD Explained by Inter-Trial (In)consistency in EEG

**DOI:** 10.3389/fpsyg.2019.01177

**Published:** 2019-06-04

**Authors:** Marianne Latinus, Yassine Mofid, Klara Kovarski, Judith Charpentier, Magali Batty, Frédérique Bonnet-Brilhault

**Affiliations:** ^1^UMR 1253, iBrain, Université de Tours, INSERM, Tours, France; ^2^Fondation Ophtalmologique Rothschild, Unité Vision et Cognition, Paris, France; ^3^CNRS (Integrative Neuroscience and Cognition Center, UMR 8002), Paris, France; ^4^Université Paris Descartes, Sorbonne Paris Cité, Paris, France; ^5^CERPPS, Université de Toulouse, Toulouse, France; ^6^CHRU de Tours, Centre Universitaire de Pédopsychiatrie, Tours, France

**Keywords:** voice perception, variability, synchrony, autism spectrum disorder, single-trial analysis, clinical profile, subgroups

## Abstract

A relative indifference to the human voice is a characteristic of Autism Spectrum Disorder (ASD). Yet, studies of voice perception in ASD provided contradictory results: one study described an absence of preferential response to voices in ASD while another reported a larger activation to vocal sounds than environmental sounds, as seen in typically developed (TD) adults. In children with ASD, an absence of preferential response to vocal sounds was attributed to an atypical response to environmental sounds. To have a better understanding of these contradictions, we re-analyzed the data from sixteen children with ASD and sixteen age-matched TD children to evaluate both inter- and intra-subject variability. Intra-subject variability was estimated with a single-trial analysis of electroencephalographic data, through a measure of inter-trial consistency, which is the proportion of trials showing a positive activity in response to vocal and non-vocal sounds. Results demonstrate a larger inter-subject variability in response to non-vocal sounds, driven by a subset of children with ASD (7/16) who do not show the expected negative Tb peak in response to non-vocal sounds around 200 ms after the start of the stimulation due to a reduced inter-trial consistency. A logistic regression model with age and clinical parameters allowed demonstrating that not a single parameter discriminated the subgroups of ASD participants. Yet, the electrophysiologically-based groups differed on a linear combination of parameters. Children with ASD showing a reduced inter-trial consistency were younger and characterized by lower verbal developmental quotient and less attempt to communicate by voice. This data suggests that a lack of specialization for processing social signal may stem from an atypical processing of environmental sounds, linked to the development of general communication abilities. Discrepancy reported in the literature may arise from that heterogeneity and it may be inadequate to divide children with ASD based only on intellectual quotient or language abilities. This analysis could be a useful tool in providing complementary information for the functional diagnostic of ASD and evaluating verbal communication impairment.

## Introduction

Autism Spectrum Disorder (ASD) is a neurodevelopmental disorder characterized by (1) impaired communication and social interaction and, (2) repeated and restricted patterns of behaviors (American Psychiatric Association [APA], 2013). The first dimension of ASD describes atypicalities in social communication including a lack of response to first names and a lack of interest in the human voice (Kanner, 1943; Klin, 1991; Ceponiene et al., 2003), which could stem from impaired voice perception. The second dimension of ASD mainly relates to non-social behavior, however, it also describes peculiarities linked to atypical voice perception and/or production such as echolalia and hyper/hypo sensitivity to auditory stimulation, and to voice in particular (American Psychiatric Association [APA], 2013).

Voices have been shown to yield preferential brain responses over a range of non-vocal sounds in both children and adults (Belin et al., 2000; von Kriegstein et al., 2003). A larger response to vocal than non-vocal sounds is observed with functional Magnetic Resonance Imaging (fMRI) in regions located along the superior temporal sulcus (STS); these functional regions have been named Temporal Voice Areas (TVA; Belin et al., 2000; Pernet et al., 2015). Bilateral TVAs were described in 94% of a large sample of typically developed (TD) adults (Pernet et al., 2015). This sensitive response to vocal sounds is present very early during typical development, with the first description of TVA at about 5 months old (Belin and Grosbras, 2010; Grossmann et al., 2010; Blasi et al., 2011). The activation of the TVA induces a fronto-temporal positivity to voice (FTPV; Rogier et al., 2010) over the scalp (Charest et al., 2009; Capilla et al., 2012). The FTPV was first described in a sample of children aged between 4 and 6 years old (Rogier et al., 2010). In children, the temporal response to non-vocal sounds consists of three successive deflections, negative–positive–negative (Na, Ta, Tb; Bruneau et al., 1997; Shafer et al., 2011; Bruneau et al., 2015). The FTPV overlaps the Ta and Tb responses, and the negative Tb peak appears reduced, e.g., it is less negative than the Tb recorded to non-vocal sounds (Rogier et al., 2010).

Studies on voice perception in ASD are scarce and provide contradictory results. An initial fMRI study reported no TVA activation in a small sample of adults with ASD with normal intelligence but impaired verbal fluency (Gervais et al., 2004): participants with ASD did not show a preferential response to vocal sounds. They showed a decreased activity in response to vocal sounds with respect to the TD participants, while there was no group difference in response to environmental sounds (Gervais et al., 2004). More recently, TVA were evidenced in a group of participants with ASD with normal or above normal intelligence; all participants with autism displayed a larger activity to vocal than non-vocal sounds, and, overall, the group did not differ from TD participants (Schelinski et al., 2016). Using electroencephalography (EEG), it was shown that children with ASD, including both children with normal intelligence and intellectual delay, presented an atypical response to environmental sounds but not to vocal sounds (Bidet-Caulet et al., 2017). Yet, in another study, an atypical response to vocal sounds was observed in children with ASD but not in adults with ASD (Charpentier et al., 2018). It should be noted that only vocal sounds were presented in that later study. Therefore, the cerebral processing of vocal and non-vocal sounds appears extremely heterogeneous in ASD.

These contradictions may stem from an increased heterogeneity at both the population level (Lenroot and Yeung, 2013; Hahamy et al., 2015) and the individual level in ASD. For example, at the population level, a large heterogeneity has been reported within the ASD population with participants showing either an increased or a decreased functional connectivity (Hahamy et al., 2015). At the individual level, an increased intra-subject variability in children/adolescents with ASD has been reported in EEG (Milne, 2011; Lefebvre et al., 2018): children/adolescents with ASD presented a larger variability of the latencies of the single-trial P100 component than TD children (Milne, 2011). Investigating individual subject’s brain responses using single-trial analysis could help us better understand this heterogeneity and identify the electrophysiological profile of children with ASD. This approach when combined with a clinical evaluation involving multiple behavioral scales could also improve our understanding of the functional profiles of children with ASD. To this aim, data published in Bidet-Caulet et al. (2017) were reanalyzed using a single-trial approach, and subsequently related to clinical scores with a logistic regression model.

## Materials and Methods

This study was carried out in accordance with the recommendations of the local ethics committee (Comité de Protection des Personnes (CPP) Tours Ouest 1), with written informed consent from all parents of the children and assent from the children, in accordance with the Declaration of Helsinki. The protocol was approved by the CPP Tours Ouest 1 (n°2006-RS).

Experimental design, set-up and EEG recordings are described in detail in Bidet-Caulet et al. (2017). Briefly, 16 children with ASD (1 girl; mean age: 8.5 years old [7–12 years old]) and 16 age-matched typically developing (TD; 1 girl; mean age: 8 years old [7–12 years old]) children participated in the study. They listened to two oddball sequences comprising human vocal (speech and non-speech human vocal sounds; note that in the rest of the manuscript vocal sounds refer to human vocalizations) and non-vocal sounds (animal vocalizations and sounds, man-made sounds, environmental sounds, etc…). In one sequence, the standards were the non-vocal sounds and deviants were vocal sounds; in the other, the standards were the vocal sounds and the deviants were the non-vocal sounds. The sequences were presented to the participants in random order. Only responses to standards are reported here. EEG was recorded while participants sat in a comfortable armchair in a dimly lit sound attenuated room, and watched a silent movie of their choice. In all TD children and 11 of the children with ASD, EEG was recorded with the means of 28 Ag/AgCl scalp electrodes. Five children with ASD tolerated the placement of only 11 electrodes (Fz, Cz, Pz, F7/F8, T7/T8, T5/T6, M1/M2). In addition, vertical eye movements were recorded from electrodes placed above and below the right eye. Impedance was kept below 10 kOhm; the EEG and electrooculogram signals were amplified with an analog band pass filter (0.3–70 Hz) and sampled at 500 Hz.

### Data Preprocessing

Data were preprocessed with EEGLab (Delorme and Makeig, 2004) working in the Matlab environment (The Mathworks^®^). Continuous EEG was epoched into 800 ms sweeps including a 100 ms pre-stimulus baseline; epochs were baseline corrected. EEG data were digitally re-referenced with respect to an average reference. The re-referenced, baseline corrected, epoched EEG data were submitted to an Independent Component Analysis (ICA) to identify and remove components corresponding to blinks and lateral eye movements. Epochs recorded in response to a standard stimulus immediately following a deviant stimulus were discarded. EEG sweeps with movement artifact were rejected manually, and digitally low-pass filtered using a 30 Hz Finite Impulse Response (FIR) filter. After rejection of the epochs containing an artifact, the average number of trials included in the analysis was 361 ± 15 and 357 ± 15 for TD children and 368 ± 12 and 353 ± 13 for children with ASD for environmental sounds and vocal sounds, respectively. Note that in the current analysis, epochs corresponding to animal vocalizations were included in the analysis as environmental sounds.

### Data Analysis

**FIGURE 1:**
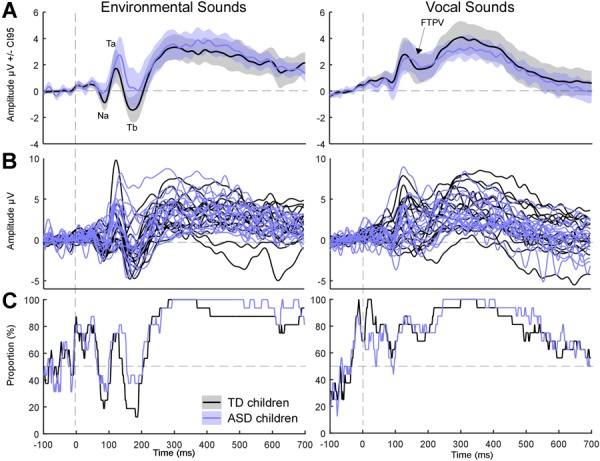
Event-related potentials for environmental and vocal sounds (on T8). Colored lines illustrate ERPs for ASD children. Black lines are for TD children. **(A)** Grand average ERPs. Shaded areas represent the 95% confidence interval built using bootstrap (*n* = 1000) with replacement of the data under H1. Note that Tb was more negative in TD children than in ASD children. **(B)** Individual ERPs. **(C)** Percentage of subjects showing a positive activity at each time point.

The analyses focused on four electrodes (T8/T7 and F8/F7) because the response to auditory stimulation is more prominent in children over these locations (Bruneau et al., 1997; Ponton et al., 2002) and because the fronto-temporal electrodes showed relevant group differences in the previous study (Bidet-Caulet et al., 2017). For each condition and each subject, an average event-related potential (ERPs) was calculated (Figure 1). To evaluate inter-subject variability, we calculated the proportion of subjects showing a positive ERPs at each time-point on T8.

**FIGURE 2:**
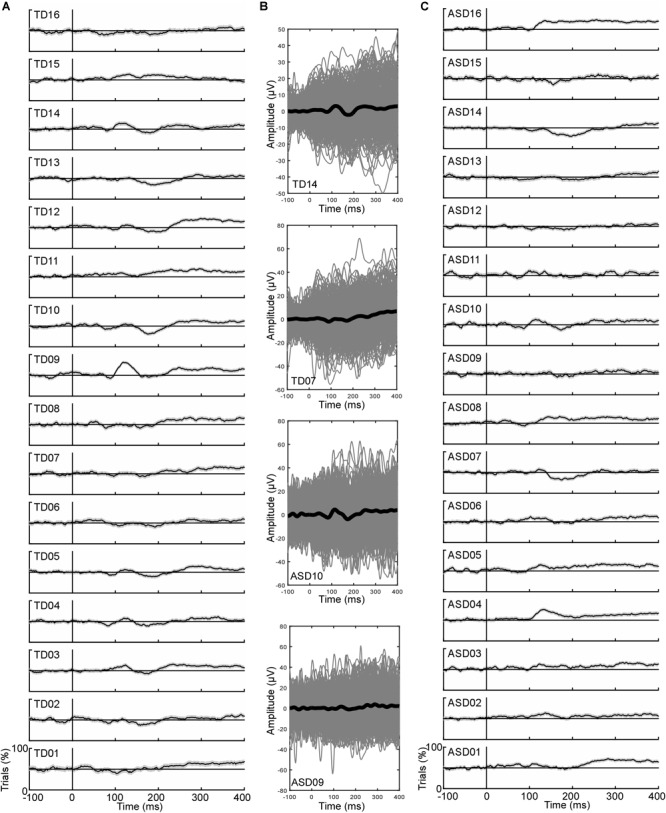
Individual data of inter-trial consistency illustrated on T8. **(A)** Proportion of trials with a positive activity in all TD children. **(B)** Illustration of the ERPs (black line) and all the trials (gray lines) for 2 TD participants and 2 ASD participants representative of “Tb-consistent” and “Tb-inconsistent” participants. **(C)** Proportion of trials with a positive activity in all ASD children. Note that no participant showed 100% of positive/negative trials at any time points highlighting the noisiness of the EEG signal.

Intra-subject variability was evaluated with a measure of inter-trial consistency under the assumption that the response evoked by all stimuli from one condition should consistently evoke the same “overall” waveform (Ouyang et al., 2016). Inter-trial consistency was estimated in each subject by measuring the proportion of trials showing a positive/negative activity at each time-point and electrode. The proportion of positive and negative (measured as a percentage of positive trials) trials were compared to chance (e.g., 50%) in the latency range of the Ta (e.g., between 90–160 ms) and Tb peaks (e.g., between 130–230 ms), respectively. In each subject, the trials were bootstrapped with replacement (*N* = 5000) to compute data-driven 95% confidence interval (CI95) around the observed proportion. The proportion of trials was deemed significantly different from chance if the upper and lower end of the CI95 were both positives for the Ta component and both negatives for the Tb component for at least 10 consecutive milliseconds, that is, if the CI95 did not include 50%. Participants were considered as “Ta-consistent” (Figure 2) if they showed a proportion of trials with a positive activity significantly different from chance in the latency range of the Ta (90–160 ms) on either electrode of each hemisphere. Similarly, participants who showed a proportion of negative trials significantly different from chance in the latency range of the Tb peak (130–230 ms) on either electrode of each hemisphere were considered as “Tb-consistent.”

The previous analysis allows identifying two subgroups of participants in the ASD group based on their electrophysiological profiles (see section “Results”). A logistic regression model with subgroups (Tb-consistent/Tb-inconsistent) as the binary outcome variable and several clinical parameters as the explanatory continuous variables was used to explore the clinical relevance of these subgroups. The following explanatory variables were entered in the model: child’s chronological age, verbal (vDQ) and non-verbal developmental quotient (nvDQ), the CARS score (The childhood Autism Rating Scale; Schopler et al., 1980), scores at the Factor 1 (BSE F1) of the Behavior Summarized Evaluation Scale (BSE – Revised; Barthelemy et al., 1997) corresponding to Interaction Disorder as well as age of first words and of first steps. In addition, we included two specific items of the BSE linked to interactive behaviors through vocal (item 5; BSE 5; does not make an effort to communicate using voice and/or words; Bruneau et al., 2003) and non-vocal signal (item 6; BSE6; lack of appropriate communicative gestures and facial expression). Parameters were standardized using data of the whole group of ASD participants. Two children with missing data (one in each subgroup of the ASD group) were excluded from the analysis. The discrimination performance of the logistic regression was assessed using a leave-two-out cross-validation procedure. To do so, the logistic regression was performed by training on N-2 children, that is removing one of each group, and the resulting model was tested on the two remaining children to obtain a prediction according to the cut-off value obtained from a Receiver Operating Curve (ROC) analysis. This operation was repeated for each children pair, i.e., 48 models tested. The prediction performance of the classifier was calculated by measuring the percentage of correct classifications. Coefficients of a final model were then calculated as the mean values of coefficients from the 48 tested models in the leave-two-out cross-validation.

## Results

### Event-Related Potentials

Grand average ERPs are displayed in Figure 1A for each group of participants. Grand average ERPs to environmental sounds are characterized by the expected succession of negative, positive, negative deflections. The Na, first negative deflection is observed between 80 and 100 ms. The Ta, a positive peak, is observed between 100 and 150 ms, while the negative Tb peak is observed between 130 and 220 ms. Grand average ERPs of the TD and ASD group appear different in the non-vocal condition: children with ASD displayed an overall more positive activity starting in the latency range of the Ta, leading to an increased Ta and reduced Tb, as evaluated statistically in Bidet-Caulet et al. (2017). Grand average ERPs of the TD and ASD group completely overlap in the vocal condition, as reported in Bidet-Caulet et al. (2017). The response to vocal sounds is characterized by a long-lasting positive wave (FTPV; Figure 1A – right panel; Rogier et al., 2010). This slow positivity overlaps the auditory evoked potentials observed for environmental sounds that is the Na, Ta, and Tb peaks.

To explore inter-subject heterogeneity, we plotted the proportion of subjects showing a positive response at each time-point on T8 for each condition (Figure 1C). The FTPV, e.g., a long-lasting positivity evoked by vocal sounds, was observed in about 80% of subjects in each group. On the contrary, for environmental sounds, marked group differences were observed around the latency of the Tb peak: while up to 88% of TD children presented a negative activity at this latency only 63% of children with ASD did so. This suggests that the group difference in processing environmental sounds is driven by a subset of children with ASD (37%) who processed environmental sounds atypically. This analysis further confirms that the main difference between children with ASD and TD children arises from differences in processing environmental sounds, in particular in the latency range of the Tb (Bidet-Caulet et al., 2017).

### Inter-Trial Consistency

Figure 2 displays, for each subject, the percentage of trials showing a positive activity on T8 for the environmental sounds. Tables 1, 2 summarize the data obtained for the inter-trial (in)consistency analysis. The mean percentage of positive trials (Table 1) in response to environmental sounds in the Ta latency range (on T8) was 57 ± 2% in TD children (range: [50–81%]) and 61 ± 1.9 in children with ASD (range: [48–76%]). The mean percentage of negative trials in response to environmental sounds in the Tb latency range was 58 ± 1.4% (range: [45–69%]) in TD children and 54 ± 2.4% (range: [33–70%]) in ASD children (Table 2). Note that this analysis provides results consistent with the ERPs measured in children, as “consistent” participants showed an ERP pattern with negative, positive, negative deflections (TD14 and ASD10 on Figure 2) while “non-consistent” participants did not (TD07 and ASD09).

**Table 1:** Variability in single-trial inconsistency in the Ta latency range for each group and condition.

	Environmental sounds				Vocal sounds			
	Right hemisphere		Left hemisphere		Right hemisphere		Left hemisphere	
	T8	F8	T7	F7	T8	F8	T7	F7
TD – mean (%) ± SEM	57 ± 2	54 ± 1	48 ± 2	40 ± 1	62 ± 2	57 ± 1	51 ± 2	54 ± 1
TD – range (%)	[50–81]	[45–61]	[40–58]	[42–56]	[49–78]	[50–65]	[44–70]	[47–65]
ASD – mean (%) ± SEM	60 ± 2	55 ± 1	52 ± 1	41 ± 2	61 ± 2	55 ± 1	53 ± 1	53 ± 1
ASD – range (%)	[48–76]	[51–60]	[42–61]	[24–48]	[50–71]	[49–64]	[45–59]	[47–52]

*Maximal proportion of positive trials in the latency range of the Ta peak for each participant is averaged across groups for each condition and electrode.*

**Table 2:** Variability in single-trial inconsistency on in the Tb latency range for each group and condition.

	Environmental sounds				Vocal sounds			
	Right hemisphere		Left hemisphere		Right hemisphere		Left hemisphere	
	T8	F8	T7	F7	T8	F8	T7	F7
TD – mean (%) ± SEM	59 ± 1	60 ± 1	63 ± 1	60 ± 1	48 ± 2	54 ± 1	56 ± 1	56 ± 1
TD – range (%)	[45–69]	[51–69]	[48–69]	[48–69]	[38–60]	[45–62]	[39–65]	[49–59]
ASD – mean (%) ± SEM	54 ± 2	57 ± 1	57 ± 1	59 ± 2	49 ± 2	54 ± 1	55 ± 1	58 ± 1
ASD – range (%)	[33–70]	[50–69]	[51–74]	[52–76]	[40–62]	[47–60]	[47–65]	[48–64]

* Maximal proportion of negative trials in the latency range of the Tb peak for each participant is averaged across groups for each condition and electrode.*

We then looked at the proportion of participants showing a high level of inter-trial consistency by exploring the percentage of “Ta-consistent” and “Tb-consistent” participants in each group. Ta- and Tb-consistent participants are identified as participants presenting a significant proportion of positive/negative trials in their respective latency range on either F8 or T8 for the right hemisphere, and on either F7 or T7 for the left hemisphere.

**FIGURE 3:**
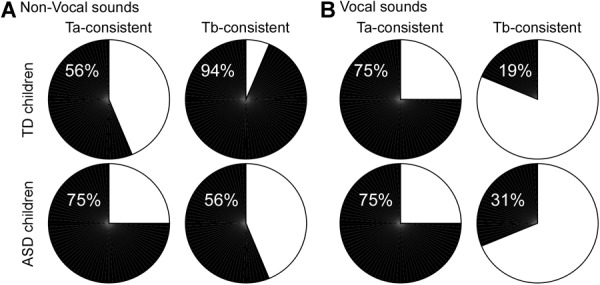
Pie chart illustration of the proportion of participants showing a high inter-trial synchronization (black) in the latency range of the Ta and Tb peaks for each group in the right hemisphere. For non-vocal **(A)** and vocal **(B)** sounds. For Ta, a high inter-trial consistency corresponds to a proportion of positive trials significantly different from 50%. For Tb, a high inter-trial consistency corresponds to a proportion of negative trials significantly different from 50%.

The proportion of “Ta-consistent” subjects in the right hemisphere in response to environmental sounds was 56% in TD children and 75% in children with ASD (Figure 2, 3A). For vocal trials, 75% of TD and ASD populations were Ta-consistent in the right hemisphere (Figure 3B). In the left hemisphere and for both conditions, less than 31% of participants in either group showed a high inter-trial consistency. Therefore, inter-trial consistency in the latency range of the Ta is larger in the right hemisphere, consistent with previous studies describing a rightward lateralization of the T-complex (Ta and Tb peak; Pang and Taylor, 2000; Shafer et al., 2011; Bruneau et al., 2015). In addition, environmental sounds more consistently yielded positive activity in the latency range of the Ta in ASD than in TD children.

The proportion of Tb-consistent subjects in response to environmental sounds in the right hemisphere was 94% in the TD group, but only 56% in the ASD group (Figure 2, 3A). In the left hemisphere, more than 80% of participants of both groups were identified as Tb-consistent for environmental sounds. Only 19% and 31% of children of the TD and ASD groups were considered Tb-consistent in response to vocal sounds in the right hemisphere, meaning that only a small number of participants showed more negative than positive trials in response to vocal sounds. This is consistent with the observation that the response to vocal sounds consists of a slow, long-lasting, positive wave, e.g., the FTPV, that overlaps the negative Tb peak (Rogier et al., 2010). However, in the left hemisphere, about 60% of children in both groups were Tb-consistent, suggesting a rightward lateralization of the FTPV.

Overall, this analysis revealed a reduced inter-trial consistency in response to non-vocal sounds in the right hemisphere in a subset (7/16; Tb-inconsistent children) of children with ASD while only 1 TD subject presented the same pattern. Note that this also means a subset of children with ASD (9/16; Tb-consistent children with ASD) displays inter-trial consistency levels similar to the TD children.

### Clinical Profiles Linked to ASD Subgroups

The electrophysiological data observed in response to environmental sounds provided evidence of two subgroups of children with ASD: the first group presented a small inter-trial consistency in the Tb-latency range, while the second group of participants showed an inter-trial consistency similar to that of TD participants. In order to optimize subgroups discrimination using both age and all clinical scores, parameters combination was performed with a logistic regression model. A leave-two-out cross validation revealed that the logistic regression model classifies the children with a 68% accuracy [95%CI: 60.6–79.6%]. The average of the parameters coefficients measured during the cross validation procedure produces the following generalized linear model as the better solution for the discrimination of the two subgroups of children with ASD in this study:

$$\begin{array}{c} Y=17.6+92.6\times \left(\mathrm{Age}\right)+40.9\times \left(\mathrm{DQv}\right)+39.3\times \left(\mathrm{DQnv}\right)+\\ 4.3\times \left(\mathrm{CARS}\right)+60\times \left(F1\right)-115.1\times \left(BSE5\right)+47.7\times \\ \left(BSE6\right)+77.6\times \left(1st\;\mathrm{words}\right)-31.2\times \left(1\mathrm{st}\;\mathrm{steps}\right) \end{array}$$

with *Y* being the combined parameter (or combined score) and F1 corresponding to the BSE factor 1.

**FIGURE 4:**
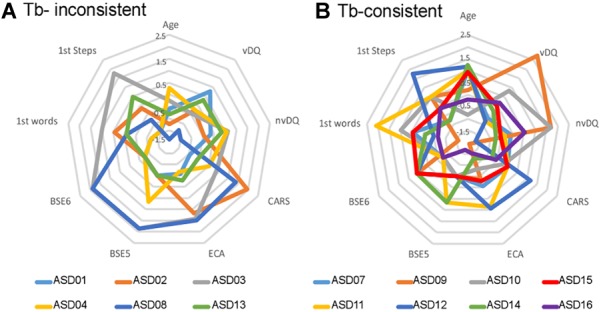
Clinical profiles of the subgroups of children with ASD. **(A)** Standardized scores observed for each child of the Tb-inconsistent subgroup. **(B)** Standardized scores observed for each child of the Tb-consistent subgroup. Age, child’s chronological age; vDQ, verbal developmental quotient; nvDQ, non-verbal developmental quotient; CARS, score at the Childhood Autism Rating Scale; first step/words: age of first steps/words; BSE-F1, Factor 1 of the Behavior Summarized Evaluation Scale; BSE5, item 5, effort toward communication using voices and/or words; BSE6, item 6, lack of facial expression and gestures. Items at the BSE are rated from 1 to 5 according to the severity of the disorder: 1 if the disorder is absent, or 5 if the symptoms are prominent and always observed. The final CARS scores is the sum of 15 categories scores; in the current study, the average (±std) CARS score was 29 (±5) so that the 0 on the radar chart represents 29.

Therefore, electrophysiological data allowed identifying two subgroups of participants with different clinical profiles based on a linear combination of all parameters (Figure 4). Children with an electrophysiological profile close to TD participants comprised mainly the older children or the young children with a higher verbal developmental quotient; they tended to show an inclination toward communication using vocal sounds (BSE5), conjointly with a later acquisition of first steps. On the other hand, “Tb-inconsistent” ASD children tended to be the younger children with a smaller verbal DQ, a higher autism severity (CARS), and a lack of endeavour toward communication (BSE5).

## Discussion

We explored inter- and intra-subject variability in already published data to better apprehend contradictions in the study of voice perception reported in the literature (Bidet-Caulet et al., 2017). Looking at inter-subject variability revealed a larger variability in response to non-vocal sounds in children with ASD, in the latency range of the Tb peak (e.g., between 130 and 200 ms). Intra-subject variability was assessed by analyzing inter-trial consistency in each subject by measuring the proportion of trials showing a positive activity in the latency range of the positive Ta peak, and the proportion of trials showing a negative activity in the latency range of the Tb peak. Single-subject analyses demonstrated reduced inter-trial consistency in children with ASD in response to environmental sounds: in 7 out of the 16 children with ASD, environmental sounds did not consistently evoke a negative activity in the latency range of the Tb peak in the right hemisphere. In contrast, only one TD subject showed a reduced inter-trial consistency. A logistic regression model highlighted that the electrophysiologically based subgroups of ASD participants could be clinically relevant. The linear combination of nine bioclinical parameters classified the autistic children in the electrophysiologically based subgroups with a 68% accuracy: the EEG signature reflects a combination of multiple bioclinical scores.

Research on voice perception in ASD has provided contradictory results. In fMRI, two studies using the same design and methodology reported either a lack of preferential response to voices (Gervais et al., 2004) or a preferential response to voices in 15 out of 16 participants with ASD (Schelinski et al., 2016). Gervais et al. (2004) described an atypical response to vocal stimulation. In EEG, Bidet-Caulet et al. (2017) reported an atypical response to environmental sounds in children with ASD. Here, we show that this effect was driven by a subset of children with ASD who do not show the expected negative Tb-peak in response to non-vocal sounds around 200 ms after the start of the stimulation (Figure 1C). Therefore, the heterogeneity of the observations regarding voice perception reported in the literature likely reflects the heterogeneity of the ASD population. Participants with ASD, without intellectual delay, present individualized patterns of functional connectivity compared to the typical template (Hahamy et al., 2015). This high heterogeneity can explain the discrepant results often reported in the literature (Hahamy et al., 2015). Therefore, it is crucial to deepen our understanding of the individual profiles of children with ASD by developing individual subject analyses and looking at intra-subject variability as was done here.

Looking at individual response patterns allowed us to highlight that heterogeneity in the ASD population was in part driven by reduced inter-trial consistency in some children with ASD. This is consistent with a recent study demonstrating a decreased inter-trial alpha-band coherence (Tallon-Baudry et al., 1996; Delorme and Makeig, 2004) in children/adolescents with ASD in the perception of visual gratings (Milne, 2011). Here, we measured inter-trial consistency in all participants by computing the proportion of trials showing a positive or negative activity in the latency range of the Ta and Tb peaks, respectively. Note that other methods than the one used here could have provided complementary information: for instance, inter-trial coherence or phase-locking factor (Makeig et al., 2002; Delorme and Makeig, 2004) are extensions of the method proposed here that take into account partial phase resetting induced by stimulus presentation, which also explains components of average ERPs (Makeig et al., 2002). Nonetheless, the method used here allows identifying different neurophysiological profiles in ASD, and future studies comparing the different methods could help understand which ones are the best suited for that purpose.

This analysis revealed that a subset of children with ASD failed to show inter-trial consistency in the latency range of the Tb peak in response to environmental sounds in the right hemisphere; yet, for the same condition, inter-trial consistency was higher in the Ta latency range. Children with ASD showed an overall more positive activity than TD children for non-vocal sounds (see Figure 3). The FTPV is a slow long-lasting positivity, originating from the TVA (Capilla et al., 2012), along the STS. The overall positivity observed at the scalp level for non-vocal sounds could reflect the activation of the STS in response to vocal, but also environmental sounds in children with ASD, traducing a lack of specialization in the processing of social signal. Consistently, because vocal sounds are processed in the STS for both children with ASD and TD children we did not observe group differences in the processing of the vocal signal.

Inter-trial consistency is a measure of the ability to synchronize neuronal activity consistently across trials. Therefore, this suggests that the variability observed in ERPs in the ASD population may reflect an inability to synchronize neuronal activity across trials. Time-frequency analysis of EEG data, including inter-trial coherence or phase-locking factor, has often been used to evaluate neural synchrony in ASD, with mixed evidence for altered synchrony in both alpha and gamma frequency bands (Milne et al., 2009; Milne, 2011; Snijders et al., 2013; Rojas and Wilson, 2014; Lefebvre et al., 2018). Here, we showed reduced inter-trial consistency by looking at the proportion of trials showing similar polarity patterns of auditory evoked potentials. This decreased inter-trial consistency could be considered as evidence of an increased intrinsic neural noise in a subset of participants with ASD, consistent with the neural noise model of autism (Simmons et al., 2009). An increased neural noise could explain why not all environmental sounds may be processed as such, which in turn could impair the specialization of the TVA in processing vocal sounds. Children with ASD characterized by a small inter-trial consistency in the latency range of the Tb peak were the younger ones and presented a clinical profile mainly characterized by a lower verbal developmental quotient, and a lack of effort toward communication, highlighting a link between the absence of specialization to process social signal in the STS and impaired abilities in the domain of communication essentially. The Tb peak has previously been described as atypical in children with a specific language impairment (SLI; Pang and Taylor, 2000; Shafer et al., 2011), although the pattern of alterations seems different from the one observed here, as these studies used only vocal sounds. Future studies comparing children with SLI and children with ASD could help disentangling effects driven by language impairment and more general verbal (including voice) communication impairments. The data presented here explains discrepancies reported in the literature and demonstrates that dividing experimental groups of children with ASD based only on intellectual quotient or language abilities might be inadequate (Silleresi, 2018).

Finally, it is important to note that the logistic regression model on a set of clinical scores allows the classification of the subjects based on their inter-trial consistency, suggesting that this measure could be used as a complementary tool in the functional diagnostic of ASD, and in the characterization of specific alteration of verbal communication. It further provides a measure that could be useful to elaborate personalized therapeutic programs and in evaluating the benefit of therapies on improvement on several aspects of verbal communication. Note that the model found here only applies to that particular small group of subjects, however, its relatively good performance showed the potential of the model that needs to be confirmed and generalized with a larger number of participants.

## Conclusion

The data presented here describes both inter- and intra-subject heterogeneity in ASD in the perception of auditory stimuli. We identify a subgroup of children with ASD with reduced inter-trial consistency. This reduced inter-trial consistency in response to non-vocal sounds, which could reflect a lack of specialization for processing social signal, may stem from an atypical processing of environmental sounds linked to the development of verbal communication abilities.

## Ethics Statement

This study was carried out in accordance with the recommendations of the Ethics Committee of the University Hospital of Tours, with written informed consent from all parents of the children and assent from the children. All subjects gave written informed consent in accordance with the Declaration of Helsinki. The protocol was approved by the Comité de Protection des Personnes (CPP) Tours Ouest 1 (n°2006-RS).

## Author Contributions

ML conceived, analyzed, and interpreted the data, and wrote the manuscript. YM, JC, and KK contributed to the data analysis. MB and FB-B contributed to the data interpretation. All authors contributed in revising the manuscript critically for important intellectual content, provided approval for publication of the content, and agreed to be accountable for all aspects of the work.

## Conflict of Interest Statement

The authors declare that the research was conducted in the absence of any commercial or financial relationships that could be construed as a potential conflict of interest.
